# Peroxidation of n-3 Polyunsaturated Fatty Acids Inhibits the Induction of iNOS Gene Expression in Proinflammatory Cytokine-Stimulated Hepatocytes

**DOI:** 10.1155/2011/374542

**Published:** 2011-06-07

**Authors:** Yoshiro Araki, Miho Matsumiya, Takashi Matsuura, Masaharu Oishi, Masaki Kaibori, Tadayoshi Okumura, Mikio Nishizawa, Hideho Takada, A-Hon Kwon

**Affiliations:** ^1^Department of Surgery, Kansai Medical University, 10-15 Fumizonocho, Moriguchi, Osaka 570-8506, Japan; ^2^The Research Organization of Science and Technology, Ritsumeikan University, Kusatsu, Shiga 525-8577, Japan; ^3^Department of Biomedical Sciences, College of Life Sciences, Ritsumeikan University, Kusatsu, Shiga 525-8577, Japan

## Abstract

Eicosapentaenoic acid and docosahexaenoic acid (EPA/DHA), n-3 polyunsaturated fatty acids (PUFAs), have a variety of biological activities including anti-inflammatory and anticancer effects. We hypothesized that their peroxidized products contributed in part to anti-inflammatory effects. In the liver, the production of nitric oxide (NO) by inducible nitric oxide synthase (iNOS) has been implicated as one of the factors in hepatic inflammation and injury. We examined whether the peroxidation of EPA/DHA influences the induction of iNOS and NO production in proinflammatory cytokine-stimulated cultured hepatocytes, which is *in vitro* liver inflammation model. Peroxidized EPA/DHA inhibited the induction of iNOS and NO production in parallel with the increased levels of their peroxidation, whereas unoxidized EPA/DHA had no effects at all. Peroxidized EPA/DHA reduced the activation of transcription factor, NF-**κ**B, and the expression of the iNOS antisense transcript, which are involved in iNOS promoter transactivation (mRNA synthesis) and its mRNA stabilization, respectively. These findings demonstrated that peroxidized products of EPA/DHA suppressed the induction of iNOS gene expression through both of the transcriptional and posttranscriptional steps, leading to the prevention of hepatic inflammation.

## 1. Introduction

Accumulated evidence indicates that eicosapentaenoic acid and docosahexaenoic acid (EPA/DHA), which are n-3 polyunsaturated fatty acids (PUFAs) and are abundant in fish oil, have a variety of biological activities such as antioxidative, anti-inflammatory, and anticancer effects. We previously reported the anti-inflammatory and anticancer effects of various PUFAs both *in vivo* and *in vitro* [1, 2]. However, the underlying mechanisms remain to be elucidated.

Since n-3 PUFAs are peroxidized spontaneously in the air, it cannot negate the possibility that their peroxidized products are involved partly in anti-inflammatory and anticancer effects. In fact, several studies demonstrated that the anticancer effect of PUFAs depended on their peroxidized levels [3–5]. Sethi [6] reported that anti-inflammatory effects of n-3 PUFAs were due to their peroxidized products. Sethi et al. [7] showed that peroxidized EPA diminished the upregulation of endothelial cell adhesion molecules such as VCAM-1 and ELAM-1 through the inhibition of NF-*κ*B activation in lipopolysaccharide- (LPS-) or cytokine-stimulated human umbilical vein endothelial cell, while unoxidized EPA had no effect. Sethi and coworkers also demonstrated that peroxidized EPA inhibited the expression of chemokines such as monocyte chemoattractant protein- (MCP-) 1 and interleukin (IL-) 8 in cytokine-stimulated endothelial cells [8, 9].

In addition to such adhesion molecules and cytokines, the production of nitric oxide (NO) by inducible nitric oxide synthase (iNOS) has been implicated as one of the factors in liver inflammation and injury. Khair-El-Din et al. [10] reported that DHA inhibited iNOS induction in interferon-*γ* plus LPS-stimulated macrophages, although they did not determine the levels of peroxidized DHA.

In animal models of liver injury caused by ischemia-reperfusion, partial hepatectomy, and endotoxin shock, the induction of iNOS and NO production is upregulated concomitantly with the production of cytokines including IL-1*β*, tumor necrosis factor-*α*, IL-6, and interferon-*γ* in the liver, as we reported previously [11–15]. In these studies, drugs showing liver-protective effects inhibited the induction of iNOS and NO production as well as the decreased production of various inflammatory mediators. Furthermore, we have found that these drugs inhibited the induction of iNOS, followed by the reduction of NO production in proinflammatory cytokine-stimulated hepatocytes [13, 16, 17], which were used as a simple *in vitro* model of liver inflammation for *in vivo* animal models. Thus, the prevention of NO production is considered to be one of the indicators of anti-inflammatory effects. In this study, we examined whether the peroxidation of EPA/DHA influences iNOS induction and NO production in the primary cultures of rat hepatocytes stimulated by IL-1*β*, and if so, what is the mechanism involved?

## 2. Materials and Methods

### 2.1. Materials

EPA and DHA (EPA/DHA) were purchased from Nacalai Tesque (Kyoto, Japan). Recombinant human IL-1*β* (2 ×10^7^ U/mg protein) was provided by Otsuka Pharmaceutical Co., Ltd. (Tokushima, Japan). [*γ*-^32^P] Adenosine-5′-triphosphate (ATP; −222 TBq/mmol) was obtained from DuPont-New England Nuclear Japan (Tokyo, Japan). Rats were kept at 22°C under a 12 h/12 h light/dark cycle and received food and water *ad libitum*. All animal experiments were performed in accordance with the Guidelines for the Care and Use of Laboratory Animals of the National Institutes of Health and approved by the Animal Care Committee of Kansai Medical University.

### 2.2. Primary Cultures of Hepatocytes

Hepatocytes were isolated from male Wistar strain rats (200–300 g; Charles River, Tokyo, Japan) by collagenase (Wako Pure Chemicals, Osaka, Japan) perfusion [18, 19]. Isolated hepatocytes were suspended in culture medium at 6 × 10^5^ cells/mL, seeded into 35 mm plastic dishes (2 mL/dish; Falcon Plastic, Oxnard, CA, USA), and cultured at 37°C in a CO_2_ incubator under a humidified atmosphere of 5% CO_2_ in air. The culture medium was Williams' medium E (WE) supplemented with 10% newborn calf serum, Hepes (5 mM), penicillin (100 U/mL), streptomycin (0.1 mg/mL), dexamethasone (10 nM), and insulin (10 nM). After 5 h, the medium was replaced by fresh serum- and hormone-free WE, and the cells were cultured overnight before use in experiments. The numbers of cells attached to the dishes were calculated by counting the nuclei [20] and using a ratio of 1.37 ± 0.04 nuclei/cell (mean ± SE, *n* = 7 experiments).

### 2.3. Preparation of Peroxidized EPA/DHA

EPA/DHA were diluted into dimethylsulfoxide (DMSO) at a concentration of 300 mM and stored at −80°C under N_2_ before experiments (unoxidized EPA/DHA). For peroxidation, EPA/DHA were treated under the air at 37°C with a block incubator for 1–3 days. Unoxidized or peroxidized EPA/DHA were dissolved into WE medium at a final concentration of 500 *μ*M (DMSO concentration was 20 mM). Peroxidation levels of EPA/DHA were measured by thiobarbituric acid reactive substances (TBARSs) assay, using TBARS Assay Kit (Cayman Chemical, Ann Arbor, MI, USA) according to the instruction. Peroxidation levels of EPA/DHA were expressed as malondialdehyde (MDA) concentration equivalent, which is a typical of lipid peroxidized products.

### 2.4. Treatment of Cells with EPA/DHA

On day 1, the cells were washed with fresh serum- and hormone-free WE, and incubated with IL-1*β* (1 nM) in the same medium in the presence or absence of EPA (500 *μ*M) or DHA (500 *μ*M) with various peroxidized levels. Peroxidation times and levels of EPA/DHA used are indicated in the appropriate figures and their legends.

### 2.5. Determinations of NO Production and Lactate Dehydrogenase (LDH)

Culture medium was used for measurements of nitrite (a stable metabolite of NO) to reflect NO production by the Griess method [21] and LDH activities to reflect cell viability using a commercial kit (Wako Pure Chemicals).

### 2.6. Western Blot Analysis

Total cell lysates were obtained from cultured cells as described previously [16] with minor modifications as follows. Cells (1 × 10^6^ cells/35-mm dish) were lysed in 100–200 *μ*L of solubilizing buffer (10 mM Tris-HCl, pH 7.4, containing 1% Triton X-100, 0.5% Nonidet P-40, 1 mM EDTA, 1 mM EGTA, phosphatase inhibitor cocktail (Nacalai Tesque), 1 mM phenylmethylsulfonyl fluoride (PMSF), and protease inhibitor cocktail (Roche Diagnostics, Mannheim, Germany, passed through a 26-gauge needle, allowed to stand on ice for 30 min and then centrifuged (16,000 × g for 15 min). The supernatant (total cell lysate) was mixed with sodium dodecyl sulfate-polyacrylamide gel electrophoresis (SDS-PAGE) sample buffer (final: 125 mM Tris-HCl, pH 6.8, containing 5% glycerol, 2% SDS and 1% 2-mercaptoethanol), subjected to SDS-PAGE and electroblotted onto a polyvinylidene difluoride membrane (Bio-Rad, Hercules, CA, USA). Immunostaining was performed using primary antibodies against mouse iNOS (Affinity BioReagents, Golden, CO, USA), human phospho-I*κ*B*α* (Ser32/36 (5A5); Cell Signaling, Beverly, MA, USA), human I*κ*B*α*, human I*κ*B*β*, mouse type I IL-1 receptor (IL-1RI) (Santa Cruz Biotechnology, Santa Cruz, CA, USA) and rat *β*-tubulin (internal control; Clone TUB2.1; Sigma Chemical Co., St. Louis, MO, USA), followed by visualization with an ECL blotting detection reagent (GE Healthcare Biosciences Corp., Piscataway, NJ, USA).

### 2.7. Reverse Transcriptase-Polymerase Chain Reaction (RT-PCR)

Total RNA was extracted from cultured hepatocytes using a guanidinium-phenol-chloroform method [22] with Trizol reagent (Invitrogen, Carlsbad, CA, USA) or a phenol-free, filter-based total RNA isolation kit (RNAqueous Kit; Ambion, Austin, TX, USA) according to the manufacturer's instructions, and then treated with a TURBO DNA-free Kit (Ambion) if necessary. For strand-specific RT-PCR analysis, cDNAs were synthesized from total RNA with strand-specific primers, and step-down PCR was performed using PC708 (Astec, Fukuoka, Japan), as previously described [23, 24] with minor modifications. For iNOS, IL-1RI and elongation factor-1*α* (EF; internal control) mRNAs, an oligo (dT) primer was used for RT and the primer sets 5′-CCAACCTGCAGGTCTTCGATG-3′ and 5′-GTCGATGCACAACTGGGTGAAC-3′ (257-bp product), 5′-CGAAGACTATCAGTTTTTGGAAC-3′ and 5′-GTCTTTCCATCTGAAGCTTTTGG-3′ (327-bp product), and 5′-TCTGGTTGGAATGGTGACAACATGC-3′ and 5′-CCAGGAAGAGCTTCACTCAAAGCTT-3′ (307-bp product) were used for PCR, respectively. For the antisense transcript of iNOS, the sense primer 5′-CCTTTGCCTCATACTTCCTCAGA-3′ was used for RT and the primer set 5′-ACCAGGAGGCGCCATCCCGCTGC-3′ and 5′-ATCTTCATCAAGGAATTATACACGG-3′ (211-bp product) was used for PCR. The PCR protocols for iNOS and EF, or IL-1RI were 10 cycles of (94°C, 1 min; 72°C, 2 min), 15 cycles of (94°C, 1 min; 65°C, 1 min 30 s; 72°C, 20 s), and 5, or 15 cycles of (94°C, 1 min; 60°C, 1 min 30 s; 72°C, 20 s), respectively. The PCR protocol for the antisense transcript was 10 cycles of (94°C, 1 min; 65°C, 1 min 30 s; 72°C, 20 s), 15 cycles of (94°C, 1 min; 60°C, 1 min 30 s; 72°C, 20 s), and 5 cycles of (94°C, 1 min; 55°C, 1 min 30 s; 72°C, 20 s). The amplified products were analyzed by 3% agarose gel electrophoresis with ethidium bromide, and the levels of iNOS, IL-1RI, EF, and antisense transcript were semiquantified using a UV transilluminator. The cDNAs for the rat iNOS mRNA and antisense transcript were deposited in DDBJ/EMBL/GenBank under Accession nos. AB250951 and AB250952, respectively.

### 2.8. Electrophoretic Mobility Shift Assay (EMSA)

Nuclear extracts were prepared according to Schreiber et al. [25] with minor modifications [26]. Briefly, the dishes were placed on ice, washed with Tris-HCl-buffered saline, harvested with the same buffer using a rubber policeman and centrifuged (1,840 × g for 1 min). The precipitate (2 × 10^6^ cells from two 35-mm dishes) was suspended in 400 *μ*L of lysis buffer (10 mM Hepes, pH 7.9, 10 mM KCl, 0.1 mM EDTA, 0.1 mM EGTA, 500 U/mL trasylol, 0.5 mM PMSF, and 1 mM dithiothreitol) and incubated on ice for 15 min. After addition of Nonidet P-40 (final: 0.625%), the cells were lysed by vortexing (2-3 times for 1 min each) and centrifuged (15,000 × g for 1 min). The nuclear pellet was resuspended with extraction buffer (10 mM Hepes, pH 7.9, 0.4 M NaCl, 0.1 mM EDTA, 0.1 mM EGTA, 500 U/mL trasylol, 0.5 mM PMSF, and 1 mM dithiothreitol), followed by continuous mixing for 20 min and centrifugation (15,000 × g for 5 min). Aliquots of the supernatant (nuclear extract) were frozen in liquid nitrogen and stored at −80°C until use. Binding reactions (total: 15 *μ*L) were performed by incubating nuclear extract aliquots (4 *μ*g of protein) in reaction buffer (20 mM Hepes, pH 7.9, 1 mM EDTA, 60 mM KCl, 10% glycerol and 1 mg of poly(dI-dC)) with the probe (approximately 40,000 dpm) for 20 min at room temperature. In the case of competitor assays, the nuclear extracts were preincubated in the presence of cold probes (250-fold excess) for 30 min at 4°C. The products were electrophoresed at 100 V in a 4.8% polyacrylamide gel in high ionic strength buffer (50 mM Tris-HCl, 380 mM glycine, 2 mM EDTA, pH 8.5) and the dried gels were analyzed by autoradiography. An NF-*κ*B consensus oligonucleotide (5′-AGTTGAGGGGA-CTTTCCCAGGC-3′) from the mouse immunoglobulin *κ* light chain was purchased (Promega, Madison, WI, USA) and labeled with [*γ*-^32^P] ATP and T4 polynucleotide kinase. The protein concentration was measured by the method of [27] with a binding assay kit (Bio-Rad) using bovine serum albumin as a standard.

### 2.9. Statistical Analysis

The results shown in the figures are representative of 3-4 independent experiments yielding similar findings. Differences were analyzed by the Bonferroni-Dunn test, and values of *P* < .05 were considered to indicate statistical significance.

## 3. Results

### 3.1. Peroxidized EPA/DHA Inhibit the Production of NO in Hepatocytes

**Figure 1:**
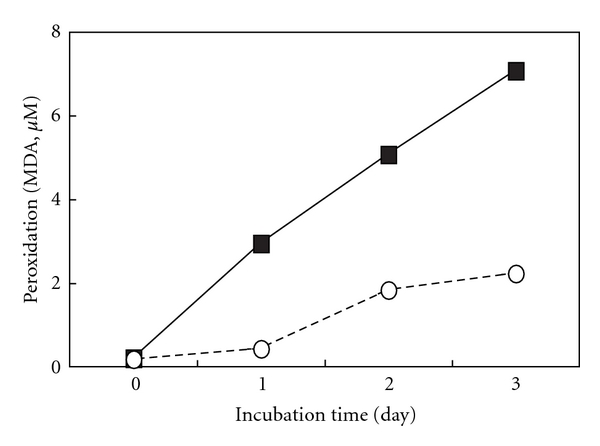
Peroxidation of EPA/DHA. EPA (open circles) and DHA (closed squares) were incubated under the air at 37°C for the indicated times. Peroxidation levels of EPA/DHA were measured by TBARS assay, and were expressed as MDA concentration equivalent per EPA/DHA (500 *μ*M). Data are means of 3 experiments.

**Figure 2:**
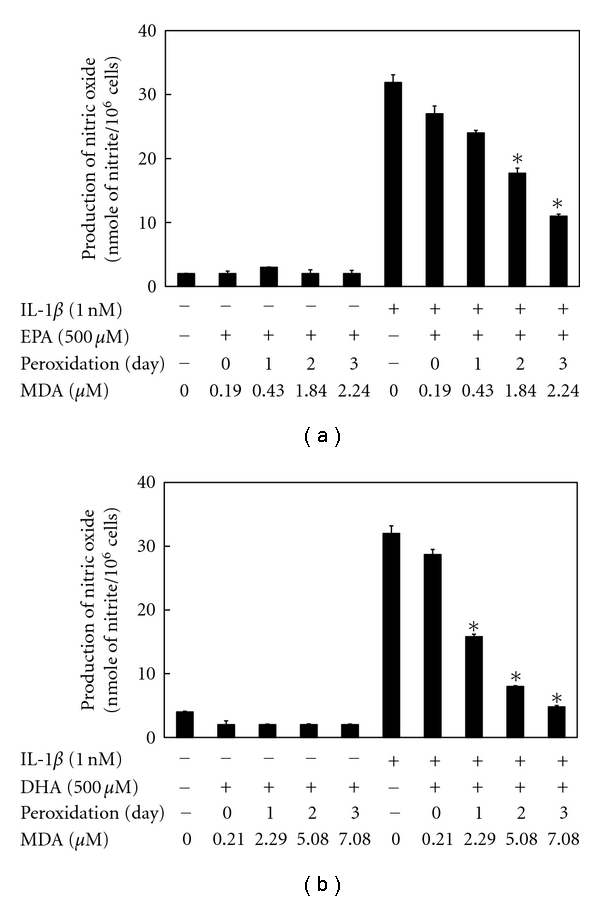
Effects of peroxidized EPA/DHA on the production of NO. Cultured hepatocytes were treated with IL-1*β* (1 nM) in the presence or absence of peroxidized EPA (a) and DHA (b) (each, 500 *μ*M) for 10 h. The levels of nitrite were measured in the culture medium (data are means ± SD for *n* = 3 dishes/point; *P < .05 versus IL-1*β* alone).

**Figure 3:**
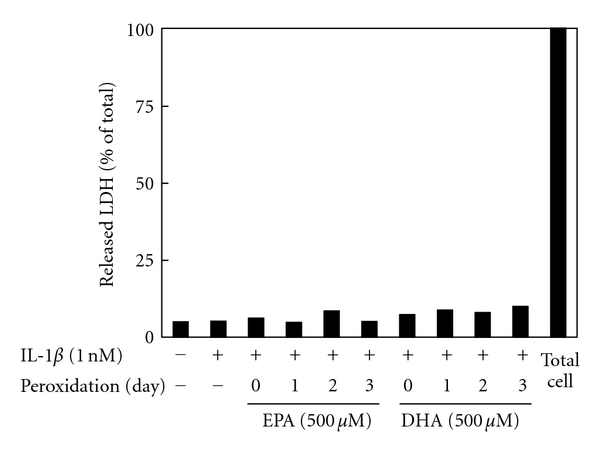
Effects of peroxidized EPA/DHA on cellular cytotoxicity. Cells were treated with IL-1*β* (1 nM) in the presence or absence of peroxidized EPA and DHA (each, 500 *μ*M) for 10 h. The LDH activities were measured in the culture medium (data are means ± SD for *n* = 3 dishes/point).

Figure 1 depicts the peroxidation levels of EPA/DHA for 1–3 days. The peroxidation levels of EPA/DHA increased to 2–7 *μ*M of MDA time dependently, which are near the ranges seen in the plasma of human (1.5–3.5 *μ*M of MDA) fed fish oil diets [8, 28]. A proinflammatory cytokine, IL-1*β*, stimulates the induction of iNOS, which is followed by the production of NO in primary cultured rat hepatocytes [29, 30]. Simultaneous addition of perox dized EPA (500 *μ*M) or DHA (500 *μ*M) with IL-1*β* decreased the levels of NO production (nitrite, NO metabolite) in peroxidation-dependent manners (Figures 2(a) and 2(b)). Neither unoxidized EPA nor DHA influenced the production of NO. Furthermore, an addition of MDA (2.5–10 *μ*M) with IL-1*β* had no effect on NO production (data not shown). Peroxidized EPA/DHA had no cellular cytotoxicity, since they showed no significant increases of released LDH in the culture medium (Figure 3) and had no effect on Trypan blue exclusion by hepatocytes (data not shown). 

**Figure 4:**
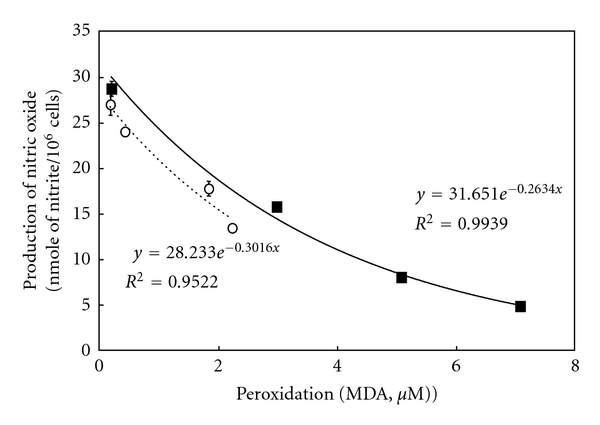
Correlation between peroxidation levels of EPA/DHA and inhibition of NO production. Cells were treated with IL-1*β* (1 nM) in the presence or absence of peroxidized EPA (open circles) and DHA (closed squares) (each, 500 *μ*M) for 10 h. Peroxidation levels of EPA/DHA, *μ*M of MDA concentration equivalent per EPA/DHA (500 *μ*M); production of NO, *n*mole of nitrite/10^6^ cells in the culture medium.

Figure 4 shows the correlation between peroxidation level of EPA/DHA and suppression of NO production. The correlations were well expressed as exponential function with high *R* ^2^ value. From these results, we obtained the equations as follows.

In case of EPA, *N* = *N*_0_ *e* ^−0.3*T*^,
*N*/*N*_0_  (NO  inhibitory  rate) = *e* ^−0.3*T*^,  *T* = 3.3 log (*N*_0_/*N*),
in case of DHA, *N* = *N*_0_ *e* ^−0.26*T*^,
*N*/*N*_0_  (NO  inhibitory  rate) = *e* ^−0.26*T*^,  *T* = 3.8 log (*N*_0_/*N*),

where *N*_0_ means NO concentration with IL-1*β*, *N* means NO concentration with IL-1*β* and peroxidized EPA/DHA, and *T* means peroxidation levels of EPA/DHA.

### 3.2. Peroxidized EPA/DHA Inhibit the Induction of iNOS in Hepatocytes

**Figure 5:**
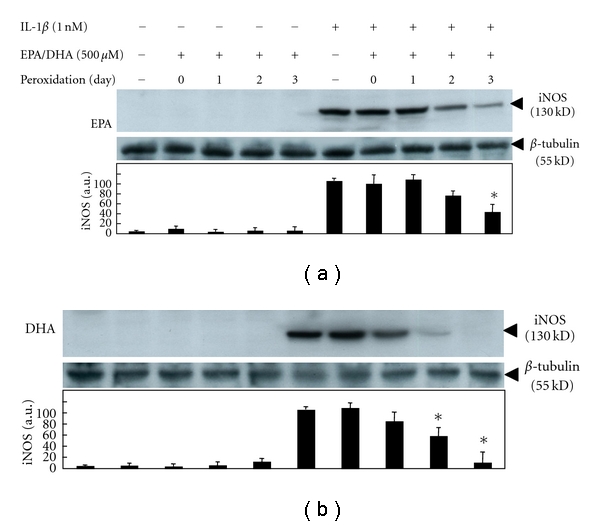
Effects of peroxidized EPA/DHA on the expression of iNOS protein. Cells were treated with IL-1*β* (1 nM) in the presence or absence of peroxidized EPA and DHA (each, 500 *μ*M) for 6 h. Cell lysates (25 *μ*g of protein) were subjected to SDS-PAGE in a 7.5% gel, and immunoblotted with an anti-iNOS or anti-*β*-tubulin antibody. The bands corresponding to iNOS were quantitated by densitometry, and the levels of iNOS were normalized by those of *β*-tubulin (lower, mean ± SD, *n* = 3 experiments; *P < .05 versus IL-1*β* alone).

**Figure 6:**
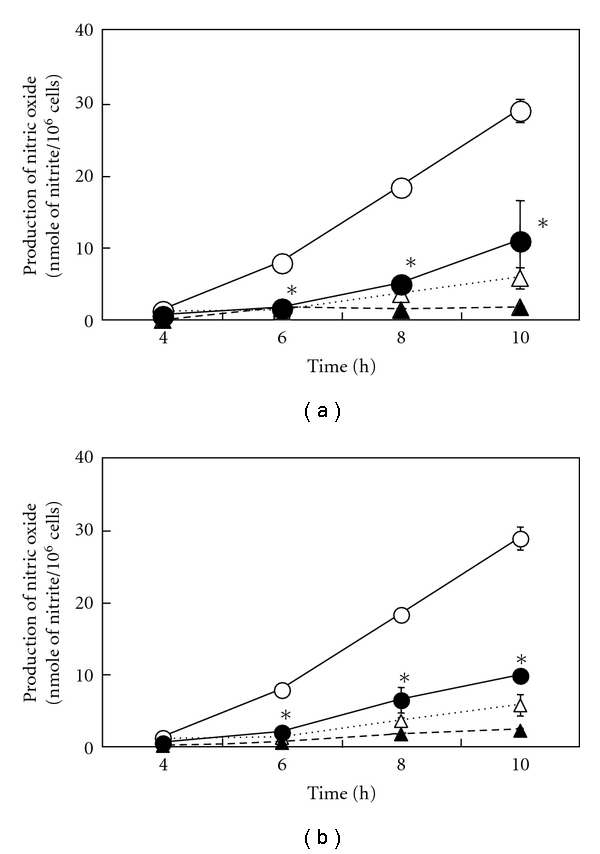
Effects of peroxidized EPA/DHA on the time course of NO production. Cells were treated with IL-1*β* (1 nM) in the presence or absence of peroxidized EPA for 3 days (a) and DHA for 1.5 days (b) (each, 500 *μ*M) for the indicated times (IL-1*β*, open circles; IL-1*β* + EPA or DHA, filled circles; EPA or DHA, filled triangles; controls (without IL-1*β* and EPA/DHA), open triangles). The levels of nitrite were measured in the culture medium (data are means ± SD for *n* = 3 dishes/point; *P < .05 versus IL-1*β* alone).

**Figure 7:**
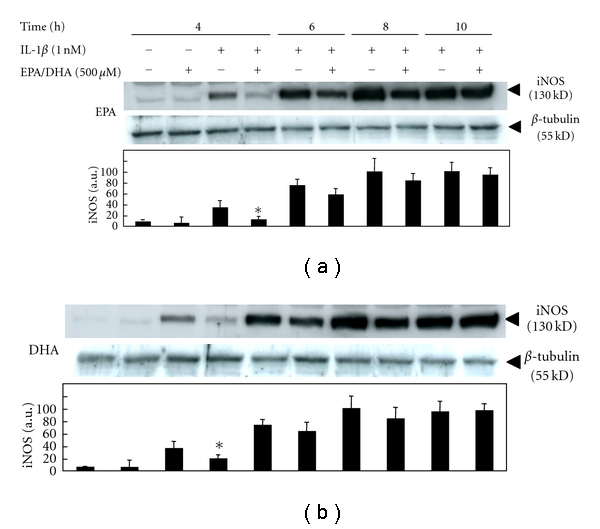
Effects of peroxidized EPA/DHA on the time course of iNOS protein expression. Cells were treated with IL-1*β* (1 nM) in the presence or absence of peroxidized EPA for 3 days and DHA for 1.5 days (each, 500 *μ*M) for the indicated times. Cell lysates (25 *μ*g of protein) were subjected to SDS-PAGE in a 7.5% gel, and immunoblotted with an anti-iNOS or anti-*β*-tubulin antibody. The bands corresponding to iNOS were quantitated by densitometry, and the levels of iNOS were normalized by those of *β*-tubulin (lower, mean ± SD, *n* = 3 experiments; *P < .05 versus IL-1*β* alone).

**Figure 8:**
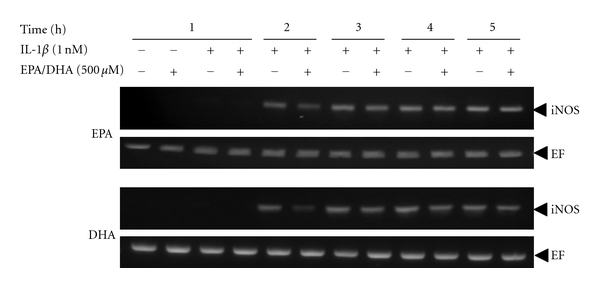
Effects of peroxidized EPA/DHA on the expression of iNOS mRNA. Cells were treated with IL-1*β* (1 nM) in the presence or absence of peroxidized EPA for 3 days and DHA for 1.5 days (each, 500 *μ*M) for the indicated times. Total RNA was analyzed by strand-specific RT-PCR to detect iNOS mRNA, using EF mRNA as an internal control.

Western blotting analysis revealed that peroxidized EPA/DHA also inhibited the expression of iNOS protein in peroxidation-dependent manners (Figure 5). Next, we examined the following experiments with peroxidized EPA for 3 days or DHA for 1.5 days, containing similar levels of MDA (approximately 2-3 *μ*M), to elucidate the mechanisms involved in their inhibitory effects on NO production. In cultured hepatocytes, the production of NO appeared at 4-5 h after IL-1*β* treatment and increased thereafter. Simultaneous addition of peroxidized EPA/DHA significantly reduced NO production time dependently (Figures 6(a) and 6(b)). Experiments with Western blotting revealed that peroxidized EPA/DHA decreased the levels of iNOS protein (Figure 7), where peroxidized EPA/DHA markedly inhibited the expression of iNOS protein at earlier time periods (4–6 h) but reduced their inhibitory effects thereafter (8–10 h). In experiments with RT-PCR, there was a similar tendency in their inhibitory effects on the expression of iNOS mRNA (Figure 8), indicating that peroxidized EPA/DHA suppressed the induction of iNOS gene expression at a transcriptional and/or posttranscriptional step.

### 3.3. Effects of Peroxidized EPA/DHA on NF-*κ*B Activation and iNOS Antisense Transcript

**Figure 9:**
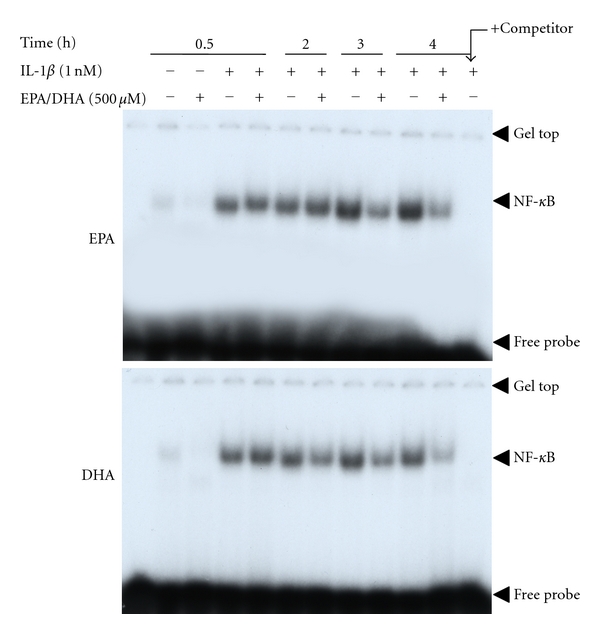
Effects of peroxidized EPA/DHA on the activation of NF-*κ*B. Cells were treated with IL-1*β* (1 nM) in the presence or absence of peroxidized EPA for 3 days and DHA for 1.5 days (each, 500 *μ*M) for the indicated times. Nuclear extracts (4 *μ*g of protein) were analyzed by EMSA.

**Figure 10:**
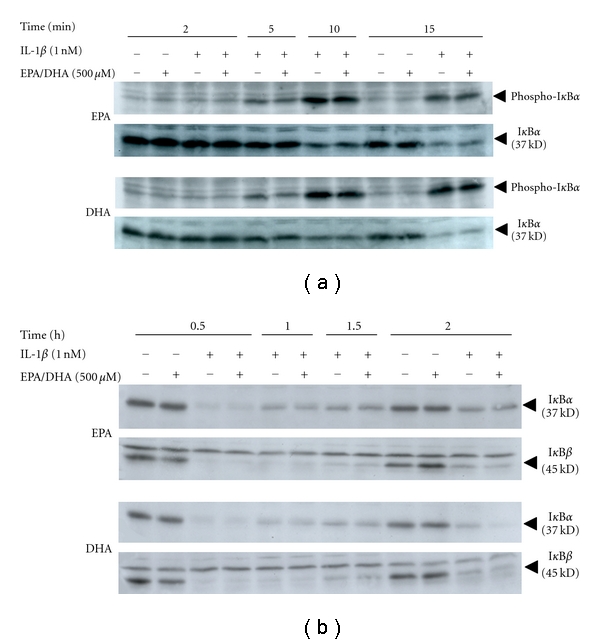
Effects of peroxidized EPA/DHA on the phosphorylation and degradation of I*κ*B proteins. Cells were treated with IL-1*β* (1 nM) in the presence or absence of peroxidized EPA for 3 days and DHA for 1.5 days (each, 500 *μ*M) for the indicated times. (a) Phosphorylation and degradation of I*κ*B*α*, and (b) degradation and recovery of I*κ*B*α* and I*κ*B*β*. Cell lysates (25 *μ*g of protein) were subjected to SDS-PAGE in a 12.5% gel, followed by immunoblotting with an anti-phospho-I*κ*B*α*, anti-I*κ*B*α* or anti-I*κ*B*β* antibody.

We examined the mechanism involved in the inhibition of iNOS mRNA expression by peroxidized EPA/DHA. IL-1*β* stimulates a rapid phosphorylation and subsequent degradation of inhibitory proteins of NF-*κ*B (I*κ*B*α* and I*κ*B*β*), followed by the activation of transcription factor NF-*κ*B. NF-*κ*B is critical for the induction of iNOS gene expression through the transactivation of iNOS promoter [16, 26]. EMSA revealed that peroxidized EPA/DHA inhibited NF-*κ*B activation (its nuclear translocation and DNA binding) (Figure 9). However, peroxidized EPA/DHA had no effects on the phosphorylation and degradation of I*κ*B*α* at earlier time periods (2–15 min) (Figure 10(a)), and the degradation and recovery of I*κ*B*α* and I*κ*B*β* at 0.5–2 h (Figure 10(b)). Alternative signal pathway in iNOS induction is the upregulation of type 1 IL-1 receptor (IL-1RI) through the activation of phosphatidylinositol 3-kinase (PI3K)/Akt [31]. Peroxidized EPA/DHA had no effect on the expression of IL-1R I protein (data not shown).

**Figure 11:**
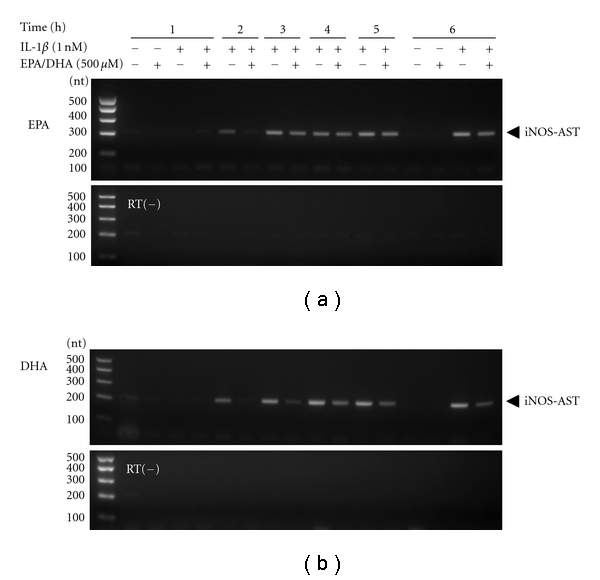
Effects of peroxidized EPA/DHA on the expression of the iNOS gene antisense transcript. Cells were treated with IL-1*β* (1 nM) in the presence or absence of peroxidized EPA for 3 days (a) and DHA for 1.5 days (b) (each, 500 *μ*M) for the indicated times. Total RNA was analyzed by strand-specific RT-PCR to detect the iNOS gene antisense transcript (iNOS-AST). RT(−): a negative control PCR using total RNA without RT.

iNOS mRNA is regulated by iNOS promoter transactivation (mRNA synthesis) through NF-*κ*B activation and by posttranscriptional modifications such as mRNA stabilization [32]. We have recently reported the expression of an iNOS gene antisense transcript that interacts with the 3′-UTR containing AU-rich elements (AREs) of iNOS mRNA and its ARE-binding proteins, thereby leading to iNOS mRNA stabilization in cytokine-stimulated hepatocytes [33]. Experiments with strand-specific RT-PCR revealed that IL-1*β* increased the expression of the iNOS gene antisense transcript in a time-dependent manner, and peroxidized EPA/DHA reduced the levels of the antisense transcript expression (Figures 11(a) and 11(b)).

## 4. Discussion

In the present study, we found that peroxidized products of EPA/DHA, but not of unoxidized EPA/DHA, inhibited iNOS induction, followed by the reduction of NO production in proinflammatory cytokine-stimulated hepatocytes (Figures 2(a) and 2(b), and 5). MDA itself did not inhibit NO production. The inhibitory effects on NO production by peroxidized EPA/DHA were dependent on their peroxidation levels (expressed as MDA equivalent) (Figure 4). Thus, it seems likely that peroxidized products of EPA/DHA, but not MDA, are involved in the inhibition of NO production. Peroxidized EPA/DHA markedly inhibited iNOS protein and its mRNA expression at earlier time periods, but less effectively thereafter (Figures 7 and 8). This observation is probably in part because of the reduction of peroxidized products by oxidoreductases such as super oxide dismutase in hepatocytes.

It is known that the induction of iNOS gene expression is regulated by the iNOS promoter transactivation and post-transcriptional modifications [32]. EMSA revealed that peroxidized EPA/DHA inhibited NF-*κ*B activation (Figure 9). NF-*κ*B typically exists in the form of p50/65 heterodimers attached to its inhibitory proteins (I*κ*Bs, I*κ*B*α*, and I*κ*B*β*) in the cytoplasm of cells. The activation of NF-*κ*B involves (i) proteolytic degradation of I*κ*Bs in proteasome after the phosphorylation by I*κ*B kinase, (ii) the translocation of NF-*κ*B to the nucleus, and (iii) its binding to the promoter *κ*B site [16]. However, peroxidized EPA/DHA did not affect the phosphorylation of I*κ*B*α* (Figure 10(a)), and the degradation and recovery of I*κ*B*α* and I*κ*B*β* (Figure 10(b)).

We previously found that the upregulation of IL-1RI through the activation of PI3K/Akt is essential for iNOS induction in addition to I*κ*B/NF-*κ*B pathway [31]. However, peroxidized EPA/DHA had no effect on the upregulation of IL-1RI protein, although they tended to reduce the levels of IL-1RI mRNA expression (data not shown). Thus, the inhibitory effect of peroxidized EPA/DHA is presumably NF-*κ*B-dependent but IL-1RI-independent mechanism. The inhibitory effect of DHA on iNOS induction seems to be superior than that of EPA due to a higher susceptibility of DHA to peroxidation.

Regarding the posttranscriptional modifications, the 3′-UTR of the iNOS mRNA in rats has six AREs (AUUU(U)A), which are associated with ARE-binding proteins such as HuR and heterogeneous nuclear ribonucleoproteins L/I (PTB), thus contributing to the stabilization of the mRNA [34]. Recently, we found that the antisense strand corresponding to the 3′-UTR of iNOS mRNA is transcribed from the iNOS gene, and that the iNOS mRNA antisense-transcript plays a key role in stabilizing the iNOS mRNA by interacting with the 3′-UTR- and ARE-binding proteins [33]. Peroxidized EPA/DHA reduced the levels of the antisense-transcript expression (Figures 11(a) and 11(b)). Taken together, peroxidized n-3 PUFAs including EPA/DHA inhibit NF-*κ*B activation and iNOS antisense transcript expression, leading to the blockades of iNOS mRNA synthesis and its stabilization, resulting in the suppression of iNOS gene induction.

When we orally take n-3 PUFA-containing foods, such as grilled or boiled fish, peroxidized products of n-3 PUFAs are significant levels in human serum [8, 28] as similar as those used in this study. Although we agree with the common view that lipid peroxidized products are considered harmful to living body, it cannot negate the possibility that peroxidized products as well as n-3 PUFAs themselves have a variety of biological activities, such as anti-inflammatory and anticancer effects.

As mentioned before, Sethi et al. reported that anti-inflammatory effects of n-3 PUFAs were due to their peroxidized products [6, 7]. Peroxidized EPA inhibited NF-*κ*B activation through PPAR*α* activation using PPAR*α* knockout mice [28] and also inhibited the expression of chemokines in cytokine-stimulated endothelial cells through the inhibition of NF-*κ*B activation via PPAR*α*-dependent pathway but not via the phosphorylation and degradation of I*κ*B*α* [9]. Similar with these observations, peroxidized EPA/DHA inhibited NF-*κ*B activation not via phosphorylation and degradation of I*κ*B*α* in our IL-1*β*-stimulated hepatocytes, while a participation of PPAR*α* remains unclear in the present study. 

Accumulating evidence indicates that NO and iNOS are concerned with cancer development. There are many reports that NO can promote cancer development through induction of DNA damage, increased angiogenesis and blood flow, prevention of apoptotic cell death, and suppression of the immune system, although there are reports that NO can suppress cancer development [35, 36]. The anticancer effect of PUFAs, including our previous reports, may be in part due to NO suppression by peroxidized products of the PUFAs. Further investigation is needed to examine whether NO suppression by peroxidized n-3 PUFAs contributes to anticancer effect.

In conclusion, peroxidized products of n-3 PUFAs suppress iNOS induction and NO production in peroxidation-dependent manners. Results suggest that peroxidized products of n-3 PUFAs are in part involved in the anti-inflammatory effects. Further, the equation from the correlation between peroxidation level and NO suppression suggests that our *in vitro* NO production model in hepatocytes may be a candidate for biological quantification of lipid peroxidation.

## References

[1] Iwamoto S, Senzaki H, Kiyozuka Y (1998). Effects of fatty acids on liver metastasis of ACL-15 rat colon cancer cells. *Nutrition and Cancer*.

[2] Senzaki H, Iwamoto S, Ogura E (1998). Dietary effects of fatty acids on growth and metastasis of KPL-1 human breast cancer cells in vivo and in vitro. *Anticancer Research*.

[3] Bégin ME, Ells G (1992). Levels of thiobarbituric acid reactive substances and the cytocidal potential of gammalinolenic and docosahexaenoic acids on ZR-75-1 and CV-1 cells. *Lipids*.

[4] Sangeetha Sagar P, Das UN, Koratkar R, Ramesh G, Padma M, Sravan Kumar G (1992). Cytotoxic action of cis-unsaturated fatty acids on human cervical carcinoma (HeLa) cells: relationship to free radicals and lipid peroxidation and its modulation by calmodulin antagonists. *Cancer Letters*.

[5] Mengeaud V, Nano JL, Fournel S, Rampal P (1992). Effects of eicosapentaenoic acid, gamma-linolenic acid and prostaglandin E on three human colon carcinoma cell lines. *Prostaglandins Leukotrienes and Essential Fatty Acids*.

[6] Sethi S (2002). Inhibition of leukocyte-endothelial interactions by oxidized omega-3 fatty acids: a novel mechanism for the anti-inflammatory effects of omega-3 fatty acids in fish oil. *Redox Report*.

[7] Sethi S, Eastman AY, Eaton JW (1996). Inhibition of phagocyte-endothelium interactions by oxidized fatty acids: a natural anti-inflammatory mechanism?. *Journal of Laboratory and Clinical Medicine*.

[8] Chaudhary A, Mishra A, Sethi S (2004). Oxidized *ω*-3 fatty acids inhibit pro-inflammatory responses in glomerular endothelial cells. *Nephron*.

[9] Mishra A, Chaudhary A, Sethi S (2004). Oxidized omega-3 fatty acids inhibit NF-*κ*B activation via a PPAR*α*-dependent pathway. *Arteriosclerosis, Thrombosis, and Vascular Biology*.

[10] Khair-El-Din T, Sicher SC, Vazquez MA (1996). Transcription of the murine iNOS gene is inhibited by docosahexaenoic acid, a major constituent of fetal and neonatal sera as well as fish oils. *Journal of Experimental Medicine*.

[11] Tsuchiya H, Kaibori M, Yanagida H (2004). Pirfenidone prevents endotoxin-induced liver injury after partial hepatectomy in rats. *Journal of Hepatology*.

[12] Tsuji K, Kwon AH, Yoshida H (2005). Free radical scavenger (edaravone) prevents endotoxin-induced liver injury after partial hepatectomy in rats. *Journal of Hepatology*.

[13] Tanaka H, Uchida Y, Kaibori M (2008). Na^+^/H^+^ exchanger inhibitor, FR183998, has protective effect in lethal acute liver failure and prevents iNOS induction in rats. *Journal of Hepatology*.

[14] Hijikawa T, Kaibori M, Uchida Y (2008). Insulin-like growth factor 1 prevents liver injury through the inhibition of TNF-*α* and iNOS induction in D-galactosamine and LPS-treated rats. *Shock*.

[15] Ishizaki M, Kaibori M, Uchida Y (2008). Protective effect of FR183998, A Na^+^/H^+^ exchanger inhibitor, and its inhibition of iNOS induction in hepatic ischemia-reperfusion injury in rats. *Shock*.

[16] Nakanishi H, Kaibori M, Teshima S (2004). Pirfenidone inhibits the induction of iNOS stimulated by interleukin-1*β* at a step of NF-*κ*B DNA binding in hepatocytes. *Journal of Hepatology*.

[17] Yoshida H, Kwon AH, Kaibori M (2008). Edaravone prevents iNOS expression by inhibiting its promoter transactivation and mRNA stability in cytokine-stimulated hepatocytes. *Nitric Oxide*.

[18] Kanemaki T, Kitade H, Hiramatsu Y, Kamiyama Y, Okumura T (1993). Stimulation of glycogen degradation by prostaglandin E in primary cultured rat hepatocytes. *Prostaglandins*.

[19] Seglen PO (1976). Preparation of isolated rat liver cells. *Methods in Cell Biology*.

[20] Horiuti Y, Ogishima M, Yano K, Shibuya Y (1991). Quantification of cell nuclei isolated from hepatocytes by cell lysis with nonionic detergent in citric acid. *Cell Structure and Function*.

[21] Green LC, Wagner DA, Glogowski J (1982). Analysis of nitrate, nitrite, and [15N]nitrate in biological fluids. *Analytical Biochemistry*.

[22] Chomczynski P, Sacchi N (1987). Single-step method of RNA isolation by acid guanidinium thiocyanate-phenol-chloroform extraction. *Analytical Biochemistry*.

[23] Nishizawa M, Nakajima T, Yasuda K (2000). Close kinship of human 20*α*-hydroxysteroid dehydrogenase gene with three aldo-keto reductase genes. *Genes to Cells*.

[24] Unezaki S, Nishizawa M, Okuda-Ashitaka E (2004). Characterization of the isoforms of MOVO zinc finger protein, a mouse homologue of Drosophila Ovo, as transcription factors. *Gene*.

[25] Schreiber E, Matthias P, Muller MM, Schaffner W (1989). Rapid detection of octamer binding proteins with ‘mini-extracts’, prepared from a small number of cells. *Nucleic Acids Research*.

[26] Oda M, Sakitani K, Kaibori M, Inoue T, Kamiyama Y, Okumura T (2000). Vicinal dithiol-binding agent, phenylarsine oxide, inhibits inducible nitric-oxide synthase gene expression at a step of nuclear factor-*κ*B DNA binding in hepatocytes. *Journal of Biological Chemistry*.

[27] Bradford MM (1976). A rapid and sensitive method for the quantitation of microgram quantities of protein utilizing the principle of protein dye binding. *Analytical Biochemistry*.

[28] Sethi S, Ziouzenkova O, Ni H, Wagner DD, Plutzky J, Mayadas TN (2002). Oxidized omega-3 fatty acids in fish oil inhibits leukocyte- endothelial interactions through activation of PPAR alpha. *Blood*.

[29] Kitade H, Sakitani K, Indue K (1996). Interleukin 1*β* markedly stimulates nitric oxide formation in the absence of other cytokines or lipopolysaccharide in primary cultured rat hepatocytes but not in Kupffer cells. *Hepatology*.

[30] Sakitani K, Kitade H, Inoue K (1997). The anti-inflammatory drug sodium salicylate inhibits nitric oxide formation induced by interleukin-1*β* at a translational step, but not at a transcriptional step, in hepatocytes. *Hepatology*.

[31] Teshima S, Nakanishi H, Nishizawa M (2004). Up-regulation of IL-1 receptor through PI3K/Akt is essential for the induction of iNOS gene expression in hepatocytes. *Journal of Hepatology*.

[32] Kleinert H, Pautz A, Linker K, Schwarz PM (2004). Regulation of the expression of inducible nitric oxide synthase. *European Journal of Pharmacology*.

[33] Matsui K, Nishizawa M, Ozaki T (2008). Natural antisense transcript stabilizes inducible nitric oxide synthase messenger RNA in rat hepatocytes. *Hepatology*.

[34] Pautz A, Linker K, Hubrich T, Korhonen R, Altenhöfer S, Kleinert H (2006). The polypyrimidine tract-binding protein (PTB) is involved in the post-transcriptional regulation of human inducible nitric oxide synthase expression. *Journal of Biological Chemistry*.

[35] Crowell JA, Steele VE, Sigman CC, Fay JR (2003). Is inducible nitric oxide synthase a target for chemoprevention?. *Molecular Cancer Therapeutics*.

[36] Lechner M, Lirk P, Rieder J (2005). Inducible nitric oxide synthase (iNOS) in tumor biology: the two sides of the same coin. *Seminars in Cancer Biology*.

